# Ethnobotanical History: Duckweeds in Different Civilizations

**DOI:** 10.3390/plants11162124

**Published:** 2022-08-15

**Authors:** Marvin Edelman, Klaus-Juergen Appenroth, K. Sowjanya Sree, Tokitaka Oyama

**Affiliations:** 1 Department of Plant and Environmental Sciences, Weizmann Institute of Science, Rehovot 7610001, Israel; 2 Plant Physiology, Matthias Schleiden Institute, University of Jena, 07743 Jena, Germany; 3 Department of Environmental Science, Central University of Kerala, Periye 671320, India; 4 Department of Botany, Graduate School of Science, Kyoto University, Kyoto 606-8502, Japan

**Keywords:** duckweed, ethnobotanical convergence, Hildegard von Bingen, Paul Emile Botta, Ritual of the Bacabs, Babylonian Talmud, Kurma Purana, Ono no Komachi, Ho Ching-ming

## Abstract

This presentation examines the history of duckweeds in Chinese, Christian, Greek, Hebrew, Hindu, Japanese, Maya, Muslim, and Roman cultures and details the usage of these diminutive freshwater plants from ancient times through the Middle Ages. We find that duckweeds were widely distributed geographically already in antiquity and were integrated in classical cultures in the Americas, Europe, the Near East, and the Far East 2000 years ago. In ancient medicinal sources, duckweeds are encountered in procedures, concoctions, and incantations involving the reduction of high fever. In this regard, we discuss a potential case of ethnobotanical convergence between the Chinese Han and Classical Maya cultures. Duckweeds played a part in several ancient rituals. In one, the unsuitability of its roots to serve as a wick for Sabbath oil lamps. In another reference to its early use as human food during penitence. In a third, a prominent ingredient in a medicinal incantation, and in a fourth, as a crucial element in ritual body purifications. Unexpectedly, it emerged that in several ancient cultures, the floating duckweed plant featured prominently in the vernacular and religious poetry of the day.

## 1. Introduction

**Figure 1:**
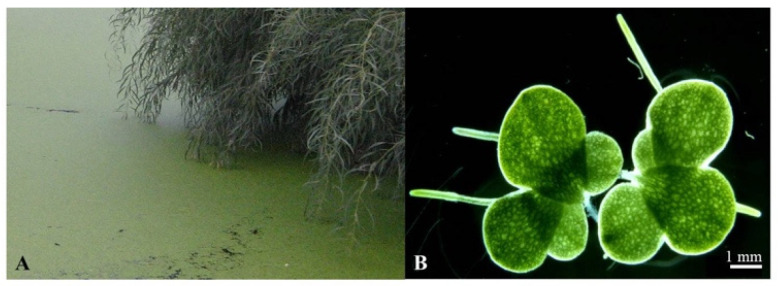
**Duckweed in nature.** (**A**) A natural population of the duckweed, *Lemna gibba* L., growing as a mat on the water surface. (**B**) Two colonies of *L. gibba* showing multiple generations of the plant vegetatively propagating. Left colony: The uppermost, large frond is the mother plant; its first daughter emerged from its left meristematic pocket and a second-generation daughter is in the process of emerging from the first daughter itself. Meanwhile, a daughter is also emerging from the mother frond’s right meristematic pocket. In this species, fronds typically have one root, several of which are seen. Photographed with illumination from below to accentuate parenchymal-cell air pockets (the lighter color areas), which generate the characteristic floating property of *Lemna* plants.

Duckweeds (Lemnaceae Martinov) are a globally spread family of higher plants with greatly reduced anatomies that float in slow-moving waters, such as found at river or lake edges or in still ponds and pools. Although these small plants are full-fledged monocot angiosperms, they reproduce mainly by vegetative budding at rapid rates, forming floating mats of verdant green in their natural habitat [1] (Figure 1).

The family is divided into five genera (*Spirodela* Schleid., *Lemna* L., *Landoltia* Les and Crawford, *Wolffia* Horkel ex Schleid. and *Wolffiella* Hegelm.) and has 36 species [2]. The first monograph dedicated to the duckweeds was published in 1839 [3], while biochemical studies of the family initiated around the 1950s [4]. Due to their miniature size, rapid growth rates, and ease of manipulation, interest in duckweeds both as a molecular-genetic research tool and in agrotechnology is now flourishing in the post-genomic era [5].

The paleontological record for the Lemnaceae is poor and is represented mainly by fossilized pollen grains from the Late Cretaceous period [6]. The paleolimnological record for Lemnaceae is also sparse, as duckweed fronds do not preserve well in lake and pond sediments [7]. Moreover, duckweeds rarely flower [1], resulting in a scarcity of pollen and seeds in sediment cores. Using, as an indicator, a highly significant association of *Lemna* with the epiphytic diatom *Lemnicola hungarica* (Grunow) Round et Basson (which does preserve well in sediments), it proved possible to detail the past presence of *Lemna minor* L. in a pond in England back a couple of centuries [8]. Aside from such ingenious attempts to go back in time, what is available to document duckweeds in antiquity are the surviving ancient manuscripts and texts.

**Figure 2:**
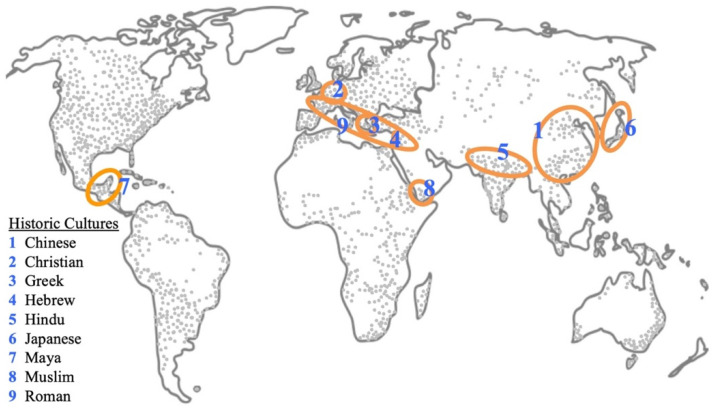
**Map of the historic cultures investigated by us with ethnobotanical reference to duckweeds.** The background map shows the multitude of locations of duckweed accessions [1], the great majority of which were collected in modern times. The circled areas show the locations of the classic cultures described here. While Theophrastus geographically pinpointed reference to duckweed to a local site in Greece [10], Dioscorides’ descriptions were presented in the wider context of his travels in the Roman Empire [11]. The references to duckweed in Chinese culture are for the Later Han [12] and Ming [13] dynasties and in the Hindu religious texts to the Himalayan cedar forests [14]. The other areas encircled encompass the general regions or countries where the cultural references occurred.

One occasionally comes across indirectly documented reference to duckweeds from classical times [9]. Records from those times are mostly from religious or medicinal manuscripts, the main two repositories of writings handed down through the ages. We uncovered ethnobotanical referral to duckweeds in Chinese, Christian, Greek, Hebrew, Hindu, Japanese, Maya, Muslim and Roman cultures. Here we document these passages from the ancient literature. In some cases, we delve deeply, in others somewhat less so, but at a minimum, the exact reference and literature passage is provided along with its contextual background. The findings are grouped in sections: habitat, medicinal usage, ritual rites and poetic association. Figure 2 shows the geographical spread of the different cultures which are discussed here on a background of the global distribution of collected duckweed isolates [1]. 

## 2. Duckweeds in Ancient Cultures

### Duckweed’s Habitat

#### Duckweed in Ancient Greece: Theophrastus’ Enquiry into Plants

It all starts with Theophrastus, the original classifier of plants. In his epic compendium *Peri phytōn historia* (“Enquiry in Plants” [10], also known as *Historia Plantarum* [15]), he used plant physiology, ecology, and agricultural methodology to arrange his treatise into ten books, nine of which are still extant. Theophrastus (371–287 BCE), born on the Greek island of Lesbos, composed his monumental work well over 2000 years ago. Rather than a completed work, the compendium is thought to represent his organized notes. He classified duckweed in terms of its aquatic habitat and coined the term “*lemna*” (water plant), which eventually became the base word for the family Lemnaceae. Mentored by Plato and then Aristotle, Theophrastus absorbed the cardinal importance of classification. His extensive botanical writings constitute a counterpart to Aristotle’s zoological works. Theophrastus may have been the first botanist to systematically look for and record characteristic features which distinguish one plant from another; he is often considered the Father of Botany [10].

In 1483 CE, under the patronage of the Papacy, the nine existing books of *Peri phytōn historia* were translated into Latin (*Historia plantarum*) by Theodorus Gaza of Thessalonica (c. 1400–1475), a prominent Greek Humanist and translator of the works of Aristotle and Theophrastus for the Roman church [16,17]. While Gaza was esteemed by his contemporaries, the translation of Theophrastus’ compendium was challenging, as botanical vocabulary in Latin is quite limited when compared to that in Greek. Gaza had both detractors (for his use of rare terms) and avid supporters for his translation [18].

In 1916, Sir Arthur Hort, Fellow at Trinity College, Cambridge, England, and amateur gardener and hybridist, produced an English translation of the first five books of Theophrastus’ *Peri phytōn historia*, naming it “Enquiry in Plants” [10]. Theophrastus’ compendium is a systematic study based on scientific observation, hence Hort’s translation of *‘historia’* to ‘Enquiry’ [19]. In his preface to the Enquiry [10], Hort pays tribute to the botanist Sir William Thiselton-Dyer, the third director of the Royal Botanical Gardens at Kew. So, too did the editors of Nature when Hort’s translation came out in 1917 [20]. It was Thiselton-Dyer who provided the identification of Theophrastus’ plants in the Enquiry and proofread Hort’s entire translation. However, there were some who took issue with Thiselton-Dyer botanical expertise [21]. We have chosen to concentrate on Hort’s translation and compare Gaza’s translation to it.

Book IV deals with “trees and plants special to particular districts and positions”. It starts off with a dissertation on the importance of position and climate, and continues with trees special to Egypt, shrubs special to Libya, herbs special to Asia, and the cold-seeking shrubs of Europe. The description then moves on to aquatic plants: of the Mediterranean Basin, the Atlantic Ocean, and the Persian Gulf, and continues with aquatic plants of rivers, marshes, and lakes, down to the resolution of a particular lake (Lake Copais) near Orchomenos, Greece. Book IV is completed with a discussion on the life spans of water plants (generally shorter) versus terrestrial ones (generally longer), weather-induced plant diseases, and the effects of human activities on plant life span.

Duckweed appears in Book IV, Section 10: “Plants peculiar to the lake of Orchomenos”. (adapted from Hort [10]). Comments in brackets are Hort’s; those in parentheses, are ours.

“[10.2] Now in the lake near Orchomenos grow the following trees and woody plants: willow(,) goat-willow(,) water-lily reeds [both that used for making pipes and the other kind](,) galingale(,) phleos(,) bulrush; and also ‘moon-flower’(,) **duckweed** and the plant called marestail: as for the plant called water-chickweed the greater part of it grows under water.”

“[10.3] Now of these most are familiar: the goat-willow(,) water-lily(,) ‘moon-flower’(,) **duckweed** and marestail probably grow also elsewhere, but are called by different names. Of these we must speak. The goat-willow is of shrubby habit and like the chaste-tree: its leaf resembles that leaf in shape, but it is soft like that of the apple, and downy. The bloom is like that of the abele, but smaller, and it bears no fruit. It grows chiefly on the floating islands; [for here too there are floating islands as in the marshes of Egypt, in Thesprotia, and in other lakes]...”

“[10.5] Of the plants of the lake they say that water-lily(,) sedge(,) and phleos bear fruit, and that of the sedge is black, and in size like that of the water-lily. The fruit of phleos is what is called the ‘plume,’ and it is used as a soap-powder... **Duckweed**(,) ‘moon-flower’ and marestail require further investigation.”

The Enquiry places duckweeds among lake plants that “probably also grow elsewhere but are called by different names”, acknowledging thereby, the spread of these plants geographically and awareness of them in those locations. The rareness and minute size of duckweed fruits (0.05 mm for *L. minor* [1]) may be behind Theophrastus’ comment in paragraph 10.5 when discussing fruits “Duckweed...require further investigation”.

In an archived copy of Gaza’s *Historia Plantarum* published in 1552 [15], the relevant passage to Hort’s sections [10.2] and [10.3] is a single, abbreviated account. The positionally comparable term to Hort’s “duckweed” is *“icma”,* possibly one of the rare terms that were unavoidable in the translation of Theophrastus’ botanically-rich Greek to Latin. Of interest is the appearance of the term “*lemna*” a few words further on in Gaza’s sentence.

*“... ad haec menififlora, icma, & quod ipnum appellant. Qnod enim lemna vocatur, altius mergitur in aqua.”*

*[15]*

and in English,

“... menififlora, icma, and what they call ipnum. For what is called the lemna is immersed deeper in water.”

(translated from the Latin by Susanne Kochs).

However, when compared to Hort’s translation

“... ‘moon-flower’(,) duckweed and the plant called marestail: as for the plant called water-chickweed the greater part of it grows under water.”

[10]

it becomes evident that Theophrastus did not coin the term “*lemna*” specifically for duckweed (which, characteristically, floats on the water’s surface) but rather as a general term for ‘water plants’ (in this case, one that “is immersed”, or “grows”, under water).

## 3. Medicinal Applications Involving Duckweeds in Ancient and Classical Sources

Duckweed, the archetype, and ever-present water plant, may have found its way into medicinal concoctions in ancient times more by simply “being there” than by strong medicinal benefit. Yet, a connecting thread may exist between sources, suggesting a remedial cooling effect on temperature-related maladies.

### 3.1. Duckweed in the Roman Empire: Dioscorides’ Materia Medica

Pedianos Dioscorides (40–90 CE), born in Cilicia of the Roman Empire (present-day Turkey), was a physician/pharmacologist in the Roman army. He lived three centuries after Theophrastus and was a contemporary of the naturalist Pliny, the Elder. Dioscorides wrote a five-volume treatise in Greek, *Peri hules iatrikēs* (on Medical Material), commonly known in the Western world as *de Materia Medica*. As T.A. Osbaldeston, translator and editor of a modern English version, succinctly put it [11]: “Theophrastus was the scientific botanist; Pliny produced the systematic encyclopaedia of knowledge; and Dioscorides was merely a medical botanist”. However, Dioscorides concentrated on the practical and pharmacopeial use of the plants he described. He was a hands-on botanist. In his dedication to his monumental work, he states, “I know many plants personally…by questioning the local inhabitants about each type of plant, I will attempt a different classification… I intend to assimilate things that are common knowledge and those that are somehow related so that the information will be exhaustive” [11]. As a result, Dioscorides’ descriptions were often sufficient for identification, including methods of preparation, medicinal uses, and dosages. Remarkably, his *de Materia Medica* functioned as the core of Western pharmacopeia through to the 19th century.

Book 4 of *de Materia Medica* describes “other herbs and roots”, among them, duckweed in Section 4.88.

*Phakos epi ton telmaton* (Adapted from a translation of the Greek by Susanne Kochs.)

**Lens on swamps/stagnant water bodies:** it is found on stagnant water; it is a moss with similarity to a lens; with its power it is cooling; against all phlegmon [diffuse inflammation], erisypelas [bacterial inflammation of the skin] and podagral [foot gout] it helps when it is applied alone or together with barley. It also seals hernias in children.

T.A. Osbaldeston identifies this entry as denoting duckweed and presents “lens” as describing *Spirodela polyrhiza* (L.) Schleid. and/or *L. minor* [11]. The phrase “a moss with similarity to a lens” may raise eyebrows; however, duckweeds have been compared to moss in other ancient texts [22], and mats of dense duckweed growth are termed “seed moss” by freshwater fishermen today in the American Lower Mississippi Alluvial Valley [23]. Dioscorides is the first to associate duckweed with the quality of “cooling” in a medicinal sense. While he then generalizes its application to “all phlegmon”, in other cultures [12,24], cooling is defined more specifically in terms of alleviating a fever.

In Osbaldeston’s English translation of *de Materia Medica* [11], an additional sentence is part of this section

“It is also called wild lens, or *epipteron*, the Romans call it *viperalis*, and some, *iceosmigdonos*.”

This addition is lacking in the Latin translation by Janus Antonius Saracenus in 1598 [25]. As with many ancient manuscripts, the literally dozens of translations of *de Materia Medica* over the centuries doubtlessly introduced copying errors, notes, deletions and additions.

### 3.2. The Divine Farmer’s Chinese Materia Medica Classic

The Divine Farmer’s Materia Medica Classic (*Shen Nong Ben Cao Jing*) is considered the authoritative version of Chinese herbal medical literature [12]. It was first committed to writing in the later Han dynasty (c. 200 CE) by Tao Hong-jing. Tao’s Classic underwent a convoluted, although not uncommon, evolution to reach its current form. The manuscript was lost but then carefully reconstructed from extant documents that had incorporated large tracts of the Classic into their own. Such behavior was common practice in the ancient and medieval world and without negative connotations. Many texts from the past would not be available to us today except for this practice [26]. Tao’s Classic also underwent multiple versions and rearrangements of sections by various scribes through the ages. The English translation by Shou-zhong Yang in 1998 [12] is based on the Classic edited by Cao Yuan-yu in 1987 and published by the Shanghai Science and Technology Press, Shanghai. This version of the Classic is thought to be closer to the original than any other.

The Classic tends more toward the mystical (Daoist influence) than to the practical and systematic, often suggesting the usage of herbs to achieve immortality and supernatural abilities. There are three levels of medicinal plants in the Classic: Superior, Middle, and Inferior. Superior class plants are nontoxic and bestow longevity; they are good for a person’s health. Middle-class plants modify temperament, and their prescription requires care. Inferior class plants are usually toxic and cannot be taken in large amounts or for prolonged periods without developing side effects. The plants in the Classic are also categorized as to flavors—sour, salty, sweet, bitter, or acrid. However, categorization by qualities of *qi* (literally, vapor or breath)—cold, hot, warm, and cool is a later addition to the Classic [12].

Duckweed (*Shui Ping*) is presented in the Classic as a Middle-class medicinal plant.

“***Shui Ping*** (water weed) is acrid and cold. It mainly treats fulminant heat and generalized itching, precipitates water *qi*, [helps] get over wine, promotes the growth of the beard and [head] hair, and quenches wasting thirst. Protracted taking may make the body light. Its other name is *Shui Hua* [water flower]. It grows in pools and swamps.”

(adapted from [12]; found just before comment 187)

Yang describes *Shui Ping*/*Shui Hua* as *Lemna* or *Spirodela*, adding that this medicinal is good for promoting sweating [12]. His comment emphasizes the effect of duckweed in treating explosive fever.

### 3.3. Duckweeds in Medieval Christian Europe

Knowledge of herbs and their use in medicine in the High Middle Ages is connected with the name St. Hildegard von Bingen (1098–1179 CE), author of *Causa et Curae* (Reasons and treatment of diseases), a medieval compilation of knowledge about plants, including the medicinal use of duckweeds. Hildegard was a nun and later an abbess [27]. she was officially promoted as Saint and Doctor of the Church on 10 May 2012 by Pope Benedict XVI.

Hildegard became a nun in the Benedictine cloister Disibodenberg in Rhineland-Palatinate, Germany already, at the age of fourteen. According to the basic rule of the Benedictines, *Ora et labora* (Latin terms for pray and work), she was responsible for the cloister garden. Following the order *Capitulare de villis* of Charles the Great from the year 812 CE, many cloisters established herb gardens and documented the knowledge of plants and their use. This way, Hildegard became familiar with plants. Later she was elected as Magistra, i.e., leader of the nun convent. She founded a new cloister, Rupertsberg, near Bingen at the river Rhine and became an abbess. This is where her name came from, Hildegard of Bingen. This new convent with the larger cloister herbal garden extended her prospects to learn more about plants and their use through her own practical experience. The second source of her knowledge was the rich cloister library, always a fixed part of each Benedictine cloister, including books about nature and medicine. It is assumed that Hildegard knew the book collection *Corpus Hippocraticum* from the fifth to second century BCE, which has part of texts from the physician Hippokrates of Kos of the physician school, Knidos. Furthermore, the oldest German medical book, *Lorscher Arzneibuch* (Engl. Lorscher Pharmacopoeia) from the 8th century, was for sure a part of the library. This book contains information about several hundred plants [27]. The third source of her knowledge was the far-reaching contacts that Hildegard could have due to her position as an abbess of a well-off Benedictine cloister. This way, she was in contact with contemporaneous scientific activities. As an example, she had intensive contact with a monastery in Salerno, south of Naples, Italy.

Popular knowledge about plants and their use in treating illnesses in medieval times was collected by women who normally did not know Latin. Therefore, their texts were not considered to be worth keeping and got lost over time. Hildegard of Bingen, however, was highly educated and wrote several books in Latin. One of her main works was called *Physica*, consisting of nine volumes, describing the scientific and medicinal properties of plants apart from giving information about animals and stones. The second main work is called *Causa et Curae* translated as Reasons and treatment of diseases [28]. In both books, the collected contemporary knowledge about plants was compiled, including the use of duckweed. Both books were originally compiled in the textbook *Liber subtilitatum diversarum rerum naturalum* (Book of diverse exact properties of all creations).

The following are a few remedies suggested by Hildegard von Bingen that make use of duckweeds. Hildegard recommended the application of an ointment against colic and described how to prepare this ointment using different plants. Her formula [28,29] is shown in Table 1.

The translator of *Causa et curae*, H. Schulz [28], assumed that the duckweed used was *L. minor*, which is most probably justified because it is the most common duckweed species in the area of the cloisters where Hildegard lived.

A second remedy, using duckweed, that Hildegard recommended was against precancerous indications and against colic, heart pain, and rheumatism for body detoxification and a weak immune system [30] (Table 2). This remedy was called “Wasserlinsenelixier” in German (duckweed elixir) and *Decoctum Lemnae cp.* in Latin. The abbreviation “*cp*”. stands most probably for *compositum*, indicating that other components apart from duckweeds are also involved. The protocol to prepare the elixir is pretty complicated [30,31].

This remedy is now available as “Wasserlinsenelixier” (duckweed elixir) by several producers and can be ordered via the Internet. It is recommended to activate metabolism and for detoxification of the human body. All these producers and shops stress that they strictly follow the protocols of Hildegard von Bingen.

## 4. Duckweeds in Ancient Religious Rituals

Rituals are essentially culture-dependent, leading to a spectrum of topics where duckweeds play a part. In one ancient text, we find duckweed (presumably its roots) in a discussion concerning its suitability as a wick for Sabbath oil lamps. In another, an ancient (the first?) reference to its use as human food in an act of asceticism. In a third, it features in a medicinal incantation, and in a fourth, in ritual purifications.

### 4.1. Duckweed in the Maya Civilization: Ritual of the Bacabs

The Maya civilization was, in its two periods, one of the foremost societies of Mesoamerica. The Maya Empire was centered in the tropical swamp lands of the Yucatan Peninsula (today encompassing Guatemala, Belize, and parts of Mexico, Honduras, and El Salvador). From discoveries at El Mirador [32], it appears that many of the cultural and architectural components of Maya society, such as massive stone temples and palaces built as stepped pyramids and embellished with multiple glyphs and inscriptions, were already in place in the Late Preclassic period (300 BCE to 150 CE), several centuries before the better-studied Classic period (250–900 CE). The Maya Empire famously and inexplicably collapsed twice, with most of the great stone cities abandoned at the termination of each period [33].

*Ah-men* (literally, he or she who knows) were the most important healers in Maya society during the Classic and Postclassic periods. They were shamans, thought of as intermediaries between the deities and the people, combining incantations to appease the gods while using their training and knowledge as herbalists to heal [34]. During the Spanish conquest of the Maya in the 16th century, almost all books and codices concerning Mayan gods, astronomy, and medicine were publicly burned by Franciscan missionaries in an effort to wipe out the local religious practices. A clandestine manuscript, discovered in the early 20th century, called the “Ritual of the Bacabs” is the only known surviving work containing the texts of the *ah-men* chants that accompanied medical treatments of the shamans. While the manuscript itself is from the Colonial Period, the chants use metaphors primarily associated with glyph inscriptions from the older Classic Period [35].

Duckweed (*Ixim ha*; literally, maize-water [35]) plays an important part in the incantation on manuscript pages 114–115 (Text 18). We considered three translations of this incantation into English [24,34,35]. Table 3 is adapted from [24], with annotations. The table’s footnotes explain the ethnological meanings and render the text logical in modern terms.

### 4.2. Duckweed in the Babylonian Talmud

The *Talmud* (from the Hebrew root ‘to study’) combines two ancient texts: the *Mishna*, the written version of Jewish oral law, compiled in the Land of Israel and completed around the year 200 CE, and the *Gemara*, the rabbinic interpretation of the Mishna, compiled in Babylonia (southeastern Mesopotamia between the Tigris and Euphrates rivers; present-day Iraq) and completed about 300 years later. It was edited thereafter by the *Savoraim* for an additional 40 to 187 years. The Babylonian Talmud is a monumental work consisting of over 5000 folio pages. It includes every imaginable topic, from the phases of the moon to financial investment strategy to how best to arise in the morning. Exhaustive analysis of dissenting opinions was the tool used by the Talmud for developing and then resolving issues. It operated at manifold levels, pitting multiple rabbinic sages over multiple generations and multiple locations, all against all, irrespective of time or space, to maximize discussion and arrive at a resolution. By banning time and space as limiting factors in the written discourse, the Talmud coincidentally produced what may be one of humanity’s first Big Data resources, a voluminous reservoir that continues to be mined today.

Duckweed enters the Talmud in a discussion in the Mishna concerning the suitability of various materials for use as an oil lamp on the Sabbath. It is prescribed in the Talmud that the festive Sabbath evening meal be held in “well lighted quarters”. The Talmud discusses, in detail, what constitutes an acceptable oil lamp for this purpose, a discussion that has since become a part of the traditional Sabbath evening services in the Synagogue:

“With what may we light [the Sabbath lamp], and with what may we not light it? We may not light it with a wick made of cedar-bast (*lechesh*), uncombed flax (*chosen*), floss-silk (*chalach*), or with a wick of willow-fiber (*iddan*), desert weed (*petilat ha-midbar*), or **duckweed** (*yaroka on the face of the water*) [since such wicks burn unevenly]. It may not be lighted with pitch (*zefet),* liquid wax (*shaava)*, castor oil *(shemen kik)*, nor with oil that must be burned and destroyed *(shemen s’raifa)*, or with tail fat *(alyah)*, nor with tallow *(chailev)*. Nahum of Media says: We may use melted tallow *(chailev mevushal)*. The sages, however, say: It is immaterial whether or not it is melted, it must not be used for the Sabbath lamp.”

(Mishna, Treatise Shabbat, Ch. 2; adapted from [38]).

The English translation of this text in the two leading traditional Hebrew prayer books matches closely, except for the phrase “*yaroka* on the face of the water”. *Yaroka* is translated in one as “duckweed” [38] and in the other as “seaweed” [39]. The Gemara discusses *yaroka*:

“This *yaroka*, what is its nature? If you say that it is the *yaroka* on top of the narrow channels [where water gathers and there is greenery on top], it crumbles [and a wick cannot be made from it]. However, Rav Pappa said that it is referring to the *yaroka* that accumulates on a ship [as greenery (at the water line) when a ship is stationary].” (Gemara, Tractate Shabbat, p. 20b; Translated by M.E., also the following passages. In brackets, comments by Rashi (1040–1105 CE), a leading interpreter of the Gemara).

**Figure 3:**
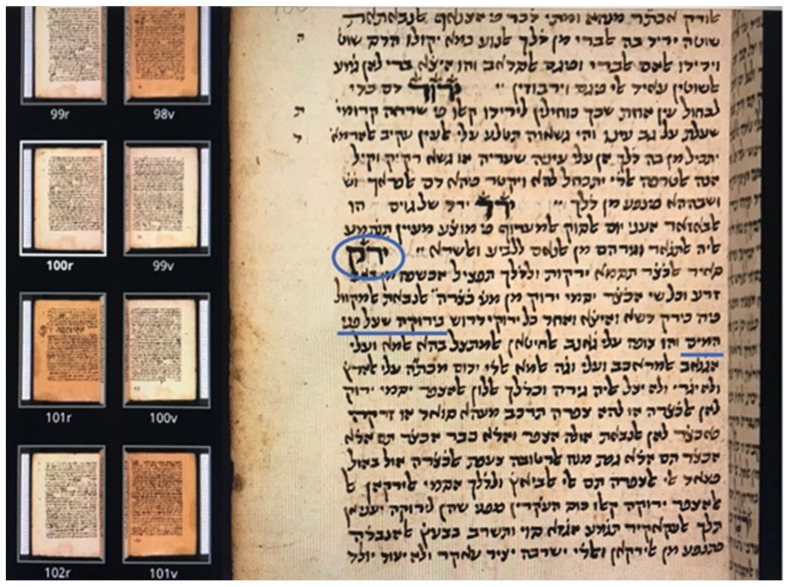
**Part of a manuscript page from Tanchum of Jerusalem’s dictionary of terms in Maimonides’ Code of Law.** Bodleian Library MS. Huntington 621, University of Oxford. [40] Manuscript date: 1393 CE. Language: Judaeo-Arabic and Hebrew. Folio page100r includes the entry for “*Yerek*” (circled in blue; literally “Greenery”). In the description of this term, Tanchum refers in Hebrew to *“yaroka* on the surface of the water” (underlined in blue), and, in Judaeo-Arabic, continues by describing this water as: “which remains on the ground without moving and is not flowing”.

The Gemara distinguishes between two types of *yaroka* based on location: one in seawater on the sides of ships and the other floating on the water in narrow channels. Rav Pappa in the Gemara favors seaweeds growing on the sides of stationary ships. If so, from where does the prayer-book designation of *yaroka* as “duckweed” arise? The answer is in the setting of the “narrow channels”, which is interpreted by other commentators as land-based, containing freshwater algae or plants [22]. Such a locale is well supported by Tanchum of Jerusalem (1219–1291 CE), author of a noted ancient dictionary (Figure 3) explaining the terms used by Maimonides (1135–1204 CE) in his *Mishne Torah* (Code of Law extracted from the Talmud). For the entry “*Yerek*” (Greenery), Tanchum states:

“... *Yaroka* on the surface of the water that remains on the ground without moving and is not flowing...”,

[40]

thus implying a stagnant, freshwater site.

**Figure 4:**
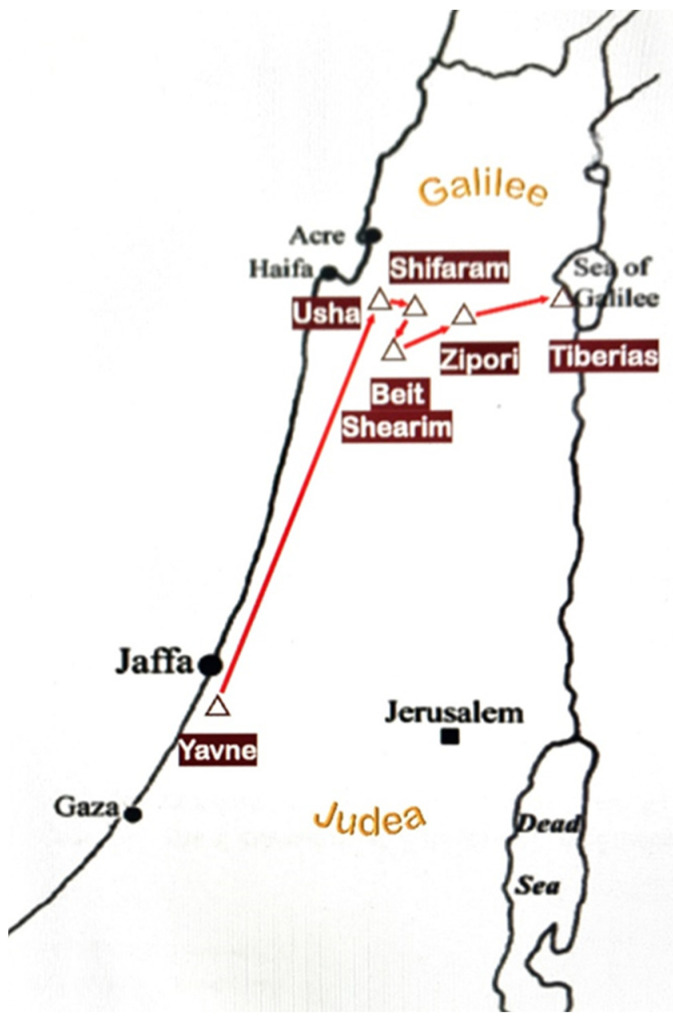
**Exile of the Sanhedrin.** To avoid restrictive edicts by the Roman Legate, the Sanhedrin (the religious and administrative leadership of the Jews at the time of the Mishna) relocated near the end of the 1st century from Yavne, a Mediterranian salt-water coastal town in Judea at the center of the country, northward to the Galilee, eventually ending up in the city of Tiberias on the shore of the freshwater Sea of Galilee by the latter part of the 2nd century.

From the text of the Talmud, it is clear that the fourth-century sages of the Gemara (such as Rav Pappa, c. 300–375 CE) were unfamiliar with the names of the materials for oil-lamp wicks described by the second-century sages of the Mishna. In addition to the passage of time, the two groups resided in different geographical locations. The sages of the Gemara flourished in exile on the shores of the Euphrates river (present-day Iraq), while the sages in our discussion of the Mishna lived and taught in the Land of Israel during the period and aftermath of the two large-scale revolts against the Roman Empire (70–200 CE). Early in this period, the center of rabbinic learning in Israel relocated northward from the town of Yavne on the shore of the Mediterranean Sea via several waystations to the city of Tiberias (Figure 4).

“...and the Sanhedrin was exiled... to Yavne, and from Yavne to Usha…and from Usha to Shefaram, and from Shefaram to Bet She’arim, and from Bet She’arim to Tzippori, and from Tzippori to Tiberias”.

(Gemara, tractate Rosh Hashana, 31a)

This relocation is significant in our context, as Tiberias sat on the shore of the Sea of Galilee, a freshwater body of about 166 km^2^ fed by the three sources of the River Jordan. (It is interesting to note that in Talmudic phraseology, “*Yam*”, the Hebrew term for “Sea”, can refer to a large body of either saline or freshwater. For example, the Mediterranean Sea was known as the “Great *Yam*” and the Sea of Galilee as the “*Yam* of Tiberias”.) Thus, in terms of geographic and ecological context, the relocation to Tiberias suggests a freshwater site for the Mishnaic discussion concerning *yaroka*.

What might be the identity of the Gemara’s freshwater *yaroka*? Filamentous algae, such as *Spirogyra*, which accumulate in freshwater ponds and are buoyed to the surface by trapped bubbles of oxygen produced during active photosynthesis in the summer, might serve [22]. So too would duckweeds from the genera *Lemna* and *Landoltia*, which float on the surface of still or slow-moving freshwater bodies and possess dangling roots of several centimeters. The “narrow channels” mentioned in the Gemara fit duckweeds well, as such channels are a favored location for finding them, particularly in rain-filled narrow troughs of ancient grain mills and wine presses at archaeological sites dotting the Galilee and Golan Heights of northern Israel (personal observation, M.E.). In this regard, the designation for the entry “*Tachlav*” in Maimonides’ glossary of drug names in his collected Medical Writings is quite germane. Maimonides, a 12th-century Pre-Renaissance polymath [41], served as a personal physician to the Sultan Salah ad-Din in Egypt, in addition to his well-known theological and philosophical pursuits. Maimonides defined the term *Tachlav* as “*yaroka* on top of the narrow channels”, which is identified in the modern edition of his Medical Writings [42] explicitly as duckweed.

### 4.3. Duckweed in Medieval Hindu Literature

The *puranas* (literally, ancients) are Hindu religious texts composed in the medieval period and written in Sanskrit. The different *puranas* contain texts on several topics, including the structure of the cosmos and the course of conduct that human beings need to follow [43]. The Sanskrit scholar, Alexander Hamilton (1762–1824), collated the *purana* manuscripts at the National Library of Paris and concluded that “after the *vedas* (literally, knowledge) texts, the *puranas* are considered the most sacred of the Indian books” [44,45]. In the past century, these Hindu texts were studied by several scholars both in India and abroad.

**Figure 5:**
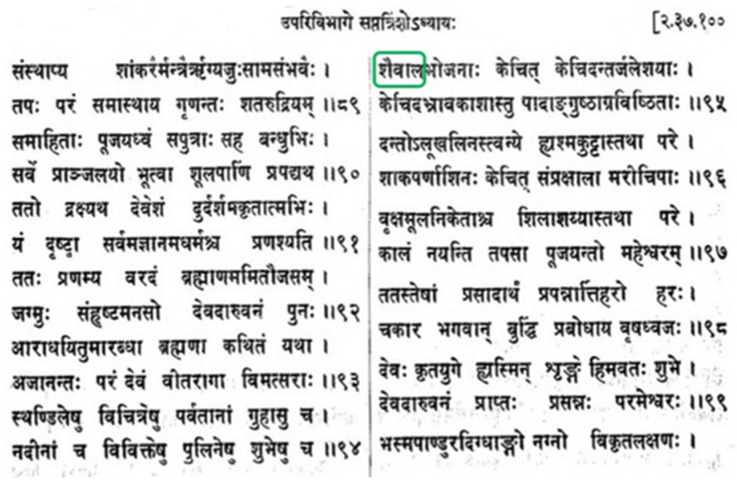
**Page 507 of the *Kurma purana*** [14]. The term *shaivaal* (alternatively, *saivala*) in Sanskrit is enclosed in the green box. It is described figuratively in [14] as “a kind of green grass-like plant growing in pools” and directly translated in [43] as duckweed.

Our focus is on the *Kurma Purana*, thought to be from the beginning of the eighth century with revisions thereafter [43]. The *Kurma purana* contains, together with other topics, teachings on acquiring knowledge by the practice of yoga and about the path to salvation. One of the stories takes place in a forest. During the course of austere penance in order to worship Lord Shiva, the sages perform several rituals, one of which is eating duckweed. Figure 5 shows an excerpt of the inscription in Sanskrit [14] referring to duckweed.

The following translation by R.H. Davis of a section of Figure 5 is adapted from [43].

“They bowed to the beneficent Brahma, unlimited in his power, and returned to the Pine (Himalayan cedar) Forest, their hearts rejoicing. They began to worship just as Brahma had advised them. Still not knowing the highest god, but without desire and without jealousy, some worshiped him on multicolored ritual platforms, some in mountain caves, and some on empty, auspicious riverbanks. Some ate **duck-weed** for food, some lay in water, and some stood on the tips of their toes, abiding amid the clouds. Others ate unground grain, or ground it with a stone. Some ate vegetable leaves, and some purified themselves by subsisting on moonbeams (rays of light [14]). Some dwelled at the foot of trees, and others made their beds upon rocks. In these ways they passed their time performing austerities and worshiping Siva.”

The translation of the Sanskrit term as per the context has been appropriately performed by Davis as duckweed [43]. This is the first historical mention of duckweed being eaten as food by human beings in the Hindu texts. Today, in-depth research has shown that these aquatic plants are a source of nutritious food for humans. Duckweeds contain high-quality protein and fatty acids [46,47] together with other phytonutrients such as phytosterols, vitamins, and minerals [48], and they do not show any adverse effects on the human system [49,50].

### 4.4. Duckweed and Ritual Purification by Yemeni Muslims

Duckweeds have a high capacity for water purification resulting from their facile ability to take up minerals and nutrients from the medium in which they grow [51]. An ancient exploitation of this property was brought to our attention several years ago by Pierre Goloubinoff, known, among other things, for his adventurous travels in Yemen [52]. Goloubinoff had read of a curious Yemini Muslim custom reported by the naturalist Paul-Emile Botta, who had been commissioned by the Natural History Museum of Paris in 1836 to explore the local flora of Yemen and collect specimens for the museum. Botta, a guest of the provincial governor, was staying at a chateau in 1837 on the flanks of mount Maammara in the province of Taiz in the southwest of the country. Nearby were some stone huts which served as housing for the families of soldiers and servants living in the governor’s castle at the mountain top. It is in this setting that Botta described a cistern used by the denizens of the stone huts [53].

… For their use and that of travelers, a large cemented cistern had been dug which received the rainwater, and in which they (the residents of the huts) not only drew their drink, but also bathed and performed their ritual purifications. … It is permitted, according to them (the residents of the huts), to wash and bathe in water which is not flowing and therefore does not renew itself, provided that it is abundant enough; while a Sunni after immersion in this way would consider himself impure, religiously speaking, as before. … the Yemenites claim, and perhaps believe, that the **duckweed** which covers the surface of stagnant waters, including their cisterns, is able to purify them (the waters), and they (the Yeminites) would not want to use standing water for purification where they would not see some (duckweed) floating. I must point out that this sect to which the inhabitants of Yemen belong is Zaydism. (translated by KSS).

**Figure 6:**
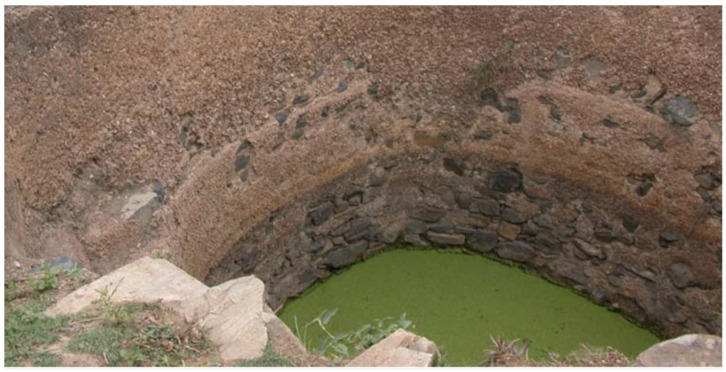
Duckweed mat covering the water surface of the cistern at Jabal éIbrahim, Himlan. From [56] with permission.

The duckweed species growing in the Taiz region is *L. minor*. While Botta was skeptical of the water cleaning powers of *Lemna*, experimental results [54,55] are on the side of the Zaydis. The driving force for the water-purifying phenomenon is the unusually rapid growth of duckweeds in nutrient-rich water (biomass doubling in about two days). Feeding ensues from the entire underside of the floating, leaf-like frond, and growth proceeds essentially exponentially, since mother fronds and then successive daughter fronds bud multiple times before aging or until crowding sets in as the water surface is covered [1]. The result is extensive depletion of many dissolved substances in the pool [54,55], which are taken up and either metabolized or stored by the duckweed, hence the clearing of the pool water. Moreover, the floating mat or moss-like cover afforded by the plant (see Figure 1A and Figure 6) retards the growth of contamination by light-seeking algae and bacteria. With careful cultivation by a local caretaker, the result is stagnant pool water that does not change its taste, color, or smell for an extended period. Botta’s singular report of stagnant pools being permitted for ablutions by Zaydi sectarians needs confirmation from additional sources and references in fiqh or fatwa.

An additional reason suggested for why the Yemeni maintain the presence of duckweed in their water sources comes from an ethnographic study of rainwater-harvesting cisterns *ad locum* in the Governorate of Hajja, Yemen [56]. Figure 6 shows a thick carpet of duckweed left in place in the cistern. As opposed to water hyacinths that increase evapotranspiration at the water surface, duckweed reduces evaporation [57]. As the natives expressed it to E. Hovden [56]: “it prevents the wind from taking the water”.

## 5. Duckweed in Ancient Secular and Religious Poetry

Duckweeds were widely spread globally already in ancient times. This gave impetus to their use as conventional imagery in poetry circles. While in Japanese culture, duckweed took on a stylized figurative meaning at the popular level, in China, its cultural imagery was habitat and biologically oriented, and so, too, its poetic context in biblical Israel.

### 5.1. Poetic Duckweed in Japanese Culture

All the references to duckweed in antiquity brought so far are from medicinal-related or religious manuscripts. Not so the history of duckweed in Japanese culture. It first appeared in the written record early in the Heian period (794–1185 CE) in collections of informal poetry and prose sponsored by the imperial court. Poetry served both political and social roles in Heian culture and became the main means of an intimate dialogue between the sexes. Much of this vernacular literature was of high quality and composed by women of the court. Remarkably, formalized poetry has remained a major means of written expression in Japanese up until modern times [58].

The Japanese islands have a mild, humid climate, and since ancient times, large areas of land have been used for rice production in paddy fields [59]. The presence of duckweed in flooded rice fields [60] was a common phenomenon in Heian-era Japan. As a result, duckweeds were familiar to the general population. *Ukikusa* (literally, floating weeds) is the term for duckweed in Japanese and is frequently found in poetry from the 9th and 10th centuries. The tiny plant became a symbol of the transience of life and mind due to its floating nature, or it was used in a rhetorical manner to evoke the feeling of melancholy or woefulness [61]. Thus, duckweeds were useful as an intermediary in the Heian poetry world and remain so in Japan today.

Ono no Komachi (born c. 850 CE) was a prominent female Heian poet. Her poems in the Kokin-waka-shu (the first and most prestigious of the imperial thirty-one-syllable waka anthologies) are mostly love poems. In Heian aristocratic society, it was impossible to function, in either public or private, without the ability to compose waka. Komachi’s poetry makes use of pivot words that have more than one meaning, allowing compression of multiple connotations within the prescribed length of the poem [58]. The best-known example involving *ukikusa* is Poem 938 (Table 4).

### 5.2. Duckweed in Classical Chinese Poetry of the Ming Dynasty

Alongside the Japanese culture of vernacular poetry, classical poetry among well-educated Chinese constituted a dominant form of social interaction up until recent times. During the Ming dynasty (1368–1644 CE), it became a conventional skill. In his excellent monograph (“The Great Recreation” [13]) on Chinese poetry of the Ming dynasty, D. Bryant focused on the life of a mid-level civil servant, Ho Ching-ming (1483–1521 CE), an Archaist poet in search of a return to an imagined ideal Chinese society of yester years. Ho passed his examination degrees, without which it was almost impossible to reach a position of influence in the civil administration [63], and spent a good part of his career years in Peking with his literary friends trying to avoid the pitfalls inherent in the politically corrupt atmosphere of the civil service. However, in 1508 and near retirement [13], Ho, together with over a hundred other officials of high character who opposed the ongoing corruption, was sacked from his post by the all-powerful and corrupt palace eunuch Liu Chin [64]. Ho then retired to his provincial village and home. This is the contextual background for Ho’s poem “Spring meditation”, translated and edited by Bryant [13], with its interesting reference to duckweed.

“The east wind comes, and in a moment the end of spring is here;

Day after day, on the clear river, I sorrow for white **duckweed**.

Toward the north, the cloudy sky is lacking any road;

From the west, over heaven and earth, haze and dust are seen.

Having known high station and low, I see how they are related,

When things come up, in safely or peril, remember the men of old…”

**Figure 7:**
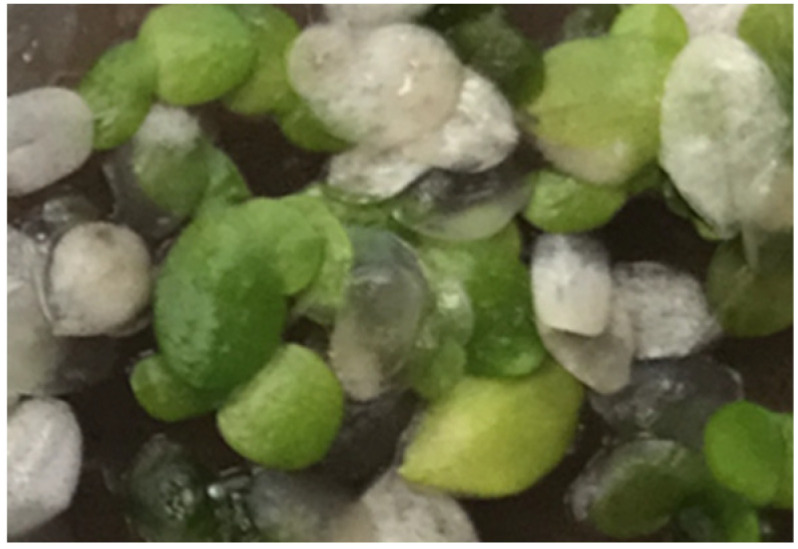
Naturally-aged white duckweed with younger green ones in a patch.

Here we understand “white duckweed” as faithfully describing aged duckweed fronds, in which chlorophyll is catabolized and the green color lost. Such naturally-aged, dead plants remain intact and visible in floating patches of live, green duckweed plants for quite some time before disintegrating (Figure 7). This is in contrast to the meaning of “white duckweed” in the Classic Maya, Ritual of the Bicabs, where the color “white” takes on a ritual and cultural meaning signifying coolness (see Table 3, footnote “h”) rather than a natural physiological meaning as here.

Ho composed several aquatic poems with duckweed imagery. For example, “On the Pond” begins with two couplets featuring duckweed, as brought by Bryant [13]

“Reeds grow at the mouth of a wintry pond;

Daily mated to the floating **duckweed**.

Breeze and ripples rock their stems;

They eddy and drift like a traveler’s roaming.”

The duckweed species with “stems” that provided poetic inspiration to Ho in this poem was most likely of the genus *Lemna*, members of which display a dangling root, with *L. aequinoctialis* and *Lemna japonica* Landolt naturally populating the Beijing area today [1].

### 5.3. Duckweed in the Hebrew Scriptures, Book of Psalms

The Book of Psalms is a collection of individual religious hymns in the Hebrew Scriptures, composed from the ninth to fifth century BCE. The hymns appear in poetic and song formation in the traditional parchment scrolls (two columns versus one for biblical prose). Their authorship is popularly attributed to King David (1040–970 BCE), who is mentioned in the titles of about half of the 150 individual Psalms [65]. However, it is quite possible that in addition to “written by David”, the meaning in Hebrew of “*l’david*” (“by”, “of,” or “to” David) in a psalm title could indicate “dedicated to”, “sung by”, “played by” David. Or, maybe all of these, as David is portrayed more than once in the Hebrew Bible as an accomplished poet–musician, a harpist, and a musical conductor.

The term “*yawvein*” is connected to watery sediments. It appears twice in the Book of Psalms but nowhere else in the Hebrew Scriptures. At the beginning of Psalm 40, *yawvein* appears following the title verse. God is praised for deliverance from some previous misfortune of the psalmist. The remaining text (not shown) of the 18-verse psalm then precedes with supplications regarding the psalmist’s present problems. (translations by M.E. based on [65,66]).

For the choir master; a Psalm, by David.

I fervently hoped for the Lord,

and He turned to me and heard my cry.

And He lifted me, from a turbulent watery dungeon,

from the mud of the ***yawvein***.

And He set my feet upon a rock,

directing my steps…

The second appearance of duckweed is at the beginning of Psalm 69, where the psalmist calls out to God in a similar fashion:

For the conductor; upon shoshanim (a musical instrument), [a Psalm] of David.

Save me O God,

for the waters have reached my neck.

I have sunk,

in the depths of ***yawvein*** there is no foothold.

I have entered deep waters,

the current is sweeping me…

Most commentators have understood *yawvein* to refer to sticky mud, a mire, or a swamp. However, the important sage, Saadia Gaon (Saadia ben Joseph Al-Fayyumi; 882–942 CE), who wrote in Judaeo-Arabic and pioneered a form of rational biblical criticism based on deep knowledge of the language of the text, translated it in Hebrew as *tachlav* [67]. As mentioned in Section 3.2, in Maimonides’ authoritative 12th-century Medical Writings [42], the term *tachlav* refers to duckweed (although filamentous algae, found in fresh and salt-water bodies, were sometimes also referred to as such [22]). In the context of Saadia Gaon’s interpretation of the unique term *yawvein*, the psalmist may have been picturing duckweed plants covering water pools or swamps so densely that they appeared as moss or carpet of grass (Figure 1A and Figure 6) and one who stepped on the “carpet” unexpectedly sank to the bottom of the waters [68].

## 6. Local Names for Duckweed in Antiquity and in the Middle Ages

The ancient local names for duckweed in the various cultures studied are listed in Table 5.

In naming duckweeds, Theophrastus concentrated on the plant’s aquatic habitat [10], Dioscorides on the characteristic lens shape of the Spirodela and Lemna fronds local to Turkey (his country of birth) and the Greek-speaking eastern Mediterranean where he was stationed [11]. The Rabbis of the Mishna focused on duckweed’s green color and freshwater location [38], the Japanese poets, its floating nature [58], the Maya [35] possibly on its ubiquitous presence as was the maize plant in their society, and the Zaydi Yemenis on its water decontamination properties.

## 7. Discussion

### A Case of Ethnobotanical Convergence?

It is of interest to note that two ancient cultures, the later Han dynasty (c. 200 CE) in eastern China and the Maya of the Classical Period (250–900 CE) in Mesoamerica, each unaware of the other, used duckweeds as a major component in their concoctions for relieving a high fever [12,24,34,35]. This can be understood in the context of natural water bodies being associated with the quality of coolness and floating duckweeds as the visible example par excellence of an aquatic plant. Yet, the match in several details (Table 6) raises the possibility that we have uncovered a putative case of ethnobotanical convergence, defined as independent origins by at least two cultures, of a given plant or family’s specific usage [72].

The Divine Farmer of the Chinese Han Dynasty, with its strong Daoistic influence, promoted duckweeds (presented as *Lemna* or *Spirodela*) as a medicinal for cooling “fulmanent heat” (eruptive fever) and “precipitating” (initiating) “water qi” (cooling strength of water) [12]. The Bicabs, with glyphs of the Maya Classical period in Mesoamerica, under the influence of the am men (shaman) healers, promoted duckweeds (presented as Lemnaceae species *L. minor* or *W. brasiliensis*) for “cooling a high fever” that “descends” (cools down) as “I seized the kinam” [35] (strength) of the “pox” (the eruptive heat) [24]. The putative case here for ethnobotanical convergence lies in the shared alleviation of a sudden or high fever by members of the family Lemnaceae and, therein, the genus *Lemna*. These points are shown in bold in Table 6. The spiritual comparisons of the Daoistic and shaman healers and their tools of trade provide some depth to the case in that the spiritual practitioners of the late Han Dynasty, like those of the Classical Maya culture, were among the most schooled healers of the period, lending an added modicum of credence to their medicinal diagnoses.

## 8. Conclusions

Our by no means exhaustive quest into the ethnobotany of duckweed in ancient cultures revealed a number of expected and unexpected references to the tiny, floating plant recorded in ancient texts, manuscripts, and glyphs. We look forward to the current presentation motivating researchers from cultures not represented here to build on the present studies. Some general points emerged. Duckweeds were widely distributed geographically already in antiquity and were integrated into classical cultures in the Americas, Europe, the Near East, and the Far East 2000 years ago. Another point that emerged is that duckweed plants infrequently served alone as a primary medicine or drug. Apparently, the plant’s strategy is to asexually outgrow the competition (mainly algae) rather than produce toxins as protectants. Yet, duckweeds appear to be of identifiable medicinal value. We described the Classic Maya and Chinese Han cultures, separated geographically and one unaware of the other, each promoting duckweed as a significant component in alleviating a high fever; possibly a novel instance of ethnobotanical convergence which needs further study. Unexpectedly, we also found that duckweeds played a role in ancient secular and religious poetry. While plant inflorescence is clearly poetically evocative, that is not the situation here. The peculiarities of tiny, floating duckweeds apparently evoked a poetic intimacy of the classical cultures with the plant itself.

## Figures and Tables

**Table 1:** Ingredients of the ointment against colic as recommended by Hildegard von Bingen.

Ingredient	Term Used by Hildegard	Plant Species	
		Latin Nomenclature	Plant Family
Feverfew	Mutterkraut	*Tanacetum parthenium* (L.) Sch.Bip.	Asteraceae
Sage	Salbei	*Salvia officinalis* L.	Lamiaceae
Zedoary	Zitwer	*Curcuma zedoaria* (Christm.) Roscoe	Zingiberaceae
Fennel	Fenchel	*Foeniculm vulgare* (L.) Mill.	Apiaceae
**Duckweed**	Wasserlinsen	*Lemna minor* L.	Lemnaceae
Erected cinquefoil	Tormentillwurzel	*Potentilla erecta* L. (Raeusch.)	Rosaceae
Charlock mustard	Senf	*Sinapis arvensis* L.	Brassicaceae
Burdock	Klette	*Arctium lappa* L.	Asteraceae

**Table 2:** Ingredients of duckweed elixir as per the protocol of Hildegard von Bingen.

Ingredient (Quantity)	Term Used by Hildegard	Plant Species		Remarks
		Latin Nomenclature	Plant Family	
**Component I: powder**				
Ginger root powder (2.5 g)	Ingwerwurzel	*Zingiber officinale* Roscoe	Zingiberaceae	Both powders were mixed
Cinnamon (10 g)	Zimtrindenpulver	*Cinnamomum verum* J.Presl	Lauraceae	
**Component II: juice**				
Sage juice from leaves (2 g)	Salbei	*Salvia officinalis* L.	Lamiaceae	All plants were homogenized, pressed out and filtered.
Fennel juice (3 g)	Fenchelkrautsaft	*Foeniculum vulgare* (L.) Mill:	Apiaceae	
Common tansy juice (2 g), without flowers, collected in spring	Rainfarnkrautsaft	*Tanacetum vulgaris* L.	Asteraceae	
**Component III: Honey wine**				
90 g Honey boiled in 1 L wine				All three components were mixed
White pepper (1.2 g)	Weisser Pfeffer	*Piper nigrum* L.	Piperaceae	
**Other components**				
**Duckweed** (20 g)	Wasserlinsen	*Lemna minor* L.	Lemnaceae	These other components were added to the three component mixture, mixed and filtered
Erected cinquefoil (40 g)	Blutwurz	*Potentilla erecta* L. (Raeusch.)	Rosaceae	
Charlock mustard (40 g)	Ackersenf	*Sinapis arvensis* L.	Brassicaceae	
Cleavers (15 g)	Labkraut	*Galium aparine* L. or *Galium verum* L.	Rubiaceae	

**Table 3:** Duckweed as the symbol of a cooling plant in the Ritual of the Bicabs **^a^**.

**This is for cooling a high fever and for cooling a pox ^b^**.
With the protecting shade of my foot, the protecting shade of my hand
I cooled the pox.
Five **^c^** are my red **^d^** hailstones **^e^**, my white hailstones, my black hailstones, my yellow hailstones.
With them I cooled the pox.
Thirteen **^c^** are the layers of my red **^d^** liturgical vestment **^f^**, my white liturgical vestment,
my black liturgical vestment, my yellow liturgical vestment.
I seized the strength **^g^** of the pox.
A black fan is my symbol when I seized the strength of the pox.
With me descends certainly my white h duckweed **^i^**.
I seized the strength of the pox.
With me descends my white **^h^** water lily.
Then it happens that I seized the strength of the pox.
Soon I will do good with the protecting shade of my foot, the protecting shade of my hand.
Amen. **^j^**

**^a^** Ritual of the Bicabs, manuscript pages 114–115. Adapted and annotated from [24].

**^b^** Title of the incantation. The term “pox”: alternatively, “eruption” [34]; “fire-pox” [35].

**^c^** Numbers 5 and 13 are significant in the complex, Classic-Maya 2-year calendar cycle. The first refers to a short, ominous period in the secular, agricultural, 365-day cycle; the second, to the number of 20-day months in the following sacred 260-day cycle [36].

**^d^** Colors are associated in the Classic Maya *materia medica* with the four cardinal directions of the world and linked to the journey of the sun deity (generator of light, time, heat, and the cardinal directions) through the sky. Red is associated with the east, where the sun rises; white with the north, from where the cooling winds of winter come; black with the west, where the sun fades and disappears; and yellow with the south, the bright broad-side of the sun [37].

**^e^** “Hailstones”, representing coldness.

**^f^** “Liturgical vestment”: alternatively, “dressing” [34]; “ornaments” [35].

**^g^** “Strength”: alternatively, “*Kinam*” [35]; “force” [34].

**^h^** “White”: representing the cooling winds of the north (see footnote “d”).

**^i^** “Duckweed”: *yxim ha* (literally, maize-water plant). It grows in the cool caves and sink holes of the Yucatan [35] and is proposed as *L. minor* or *W. brasiliensis* [24].

**^j^** “Amen”: one of the few intrusions of Christian elements in the Ritual of the Bacabs, suggesting that Maya belief had not undergone many changes by 1779, when this Colonial period manuscript was committed to writing [24].

**Table 4:** A flirtatious poem by Ono no Komachi (adapted and annotated from a translation by A. Commons in [58]).

When Fun’ya no Yasuhide became the third-ranked official of Mikawa Province and invited me to come sightseeing in the provinces, this was my reply: **^a^**	
*wabinureba* *mi o ukikusa no* *ne o taete* **^c^** *sasou mizu araba* *inamu to zo omou*	Lonely and forlorn as a **duckweed** {*uki* **^b^** *kusa*}: should flowing waters **^d^** beckon I think I’d follow.

**^a^** The headnote identifies this poem as a response of Ono no Komachi to a poem sent to her by another prominent poet of the Heian period, Fun’ya no Yasuhide.

**^b^** The “*uki*” of *ukikusa* is a pivot word meaning both “floating” (of the duckweed) and “miserable” (the poet). *Lemna aoukikusa* T.Beppu and Murata was, for a time, a synonym name (now retired) for *Lemna aequinoctialis* Welw. [62], which is a prevalent duckweed throughout most of Japan [1].

**^c^** *ne o taete* means “without a root” and often appears alongside of *ukikusa*; thus, embedding an additional subtle reference to duckweed.

**^d^** The “flowing waters” represent the message from Yasuhide. This is guided by the name of the province where Yasuhide officiated, “Mikawa”; its literal meaning is “three rivers”.

**Table 5:** Local names of duckweed in ancient cultures.

Culture	Period of History	Local Name	Literal Meaning	Ref.
Chinese	Han Dynasty, c.200 CE	***Shui Ping***	water weed	[12]
		***Shui Hua***	water flower	[12]
	Ming Dynasty, c.1500 CE	***Fu Ping***	floating duckweed	[13]
Christian	Hildegard von Bingen c.1150 CE	***Lemna***	Duckweed	[28]
Greek	Theophrastus c.330 BCE	***Lemna*** **^a^**	water plant	[10]
Hebrew	Book of Psalms c.1000 BCE	***Yawvein***	duckweed	[65,66]
		***Tachlav* ^b^**	duckweed	[42,67]
	Talmud (Mishna) c.200 CE	***Yaroka***	greenery on water	[38]
Hindu	Kurma purana c.700 CE	***Shaivaal***	weed on water ^**c**^	[69,70]
Japanese	Ono no Komachi c.850 CR	***Ukikusa*** ***Ne-nashi-k(g)usa***	floating weeds weeds without a root	[58] [58]
Maya	Ritual of the Bicab*s* c.250 CE	***Ixim ha***	maize-water plant	[35]
Roman	Dioscorides c.70 CE	***Lens***	lentil-shaped	[11]
Yemini	Zaydism c.1000 CE ^**d**^	***Simsim*** **^e^**	Sesame-seed ^**e**^	

**^a^** Theophrastus coined the Greek term “lémna”, which became the base word of the family Lemnaceae.

**^b^** Judaeo-Arabic, from the Pre-Islamic Arabic for duckweed, *al-ṭuḥlubū* [69].

**^c^** Shaivaal translated as “weed on water” by K.S.S. based on [70].

**^d^** Zaidism reached Yemen in the 11th century [71].

**^e^** Dialect term for *L. minor* in the Al-Shu‘ayb District, Al-Ḍāli‘ Governorate (Daniel Varisco, personal communication). The same term can refer to different plants and the same plant can have different names depending on the locality, even between villages. Simsim as a moniker for duckweed is likely based on sesame seed’s size and shape.

**Table 6:** Ethnobotanical convergence suggested in ancient duckweed medicinal usage.

Divine Farmer’s *Materia Medica* [12]	Ritual of the Bacabs [24,34,35]
Later Han Dynasty, eastern China (c. 200 CE)	Maya Classical Period, the Americas (250–900 CE)
**Lemnaceae**: *Lemna, Spirodela*	**Lemnaceae**: *L. minor, W*. *brasiliensis*
**“treats fulminant heat”**	**“cooling a high fever”**
Daoist influence	shaman incantation
“precipitates water *qi*”	“seized the *kinam* (strength) of the pox”

## Data Availability

All the data are available in the manuscript.
